# TKT-PARP1 axis induces radioresistance by promoting DNA double-strand break repair in hepatocellular carcinoma

**DOI:** 10.1038/s41388-023-02935-9

**Published:** 2024-01-12

**Authors:** Longpo Geng, Mingming Zhu, Dongjun Luo, Huihui Chen, Binghua Li, Yuanxiang Lao, Hongda An, Yue Wu, Yunzheng Li, Anliang Xia, Yi Shi, Zhuting Tong, Shanshan Lu, Dengqiu Xu, Xu Wang, Wenjun Zhang, Beicheng Sun, Zhu Xu

**Affiliations:** 1 Department of Hepatobiliary Surgery, Innovative Institute of Tumor Immunity and Medicine (ITIM), Anhui Province Key Laboratory of Tumor Immune Microenvironment and Immunotherapy, The First Affiliated Hospital of Anhui Medical University, Hefei, 230022 China; 2 Department of Hepatobiliary Surgery, The Affiliated Drum Tower Hospital of Nanjing University Medical School, Nanjing, 210008 China; 3 Department of Orthopedics, The Second Affiliated Hospital of Anhui Medical University, Hefei, 230061 China; 4 Department of Pharmacy, The First Affiliated Hospital of USTC (Anhui Provincial Hospital), Hefei, 230001 China; 5 School of Life Sciences, Anhui Medical University, Hefei, 230022 China; 6 Department of Burns and Plastic Surgery, Shanghai Changzheng Hospital, Shanghai, 200003 China

**Keywords:** Radiotherapy, Genomic instability

## Abstract

Hepatocellular carcinoma (HCC) stands as the fifth most prevalent malignant tumor on a global scale and presents as the second leading cause of cancer-related mortality. DNA damage-based radiotherapy (RT) plays a pivotal role in the treatment of HCC. Nevertheless, radioresistance remains a primary factor contributing to the failure of radiation therapy in HCC patients. In this study, we investigated the functional role of transketolase (TKT) in the repair of DNA double-strand breaks (DSBs) in HCC. Our research unveiled that TKT is involved in DSB repair, and its depletion significantly reduces both non-homologous end joining (NHEJ) and homologous recombination (HR)-mediated DSB repair. Mechanistically, TKT interacts with PARP1 in a DNA damage-dependent manner. Furthermore, TKT undergoes PARylation by PARP1, resulting in the inhibition of its enzymatic activity, and TKT can enhance the auto-PARylation of PARP1 in response to DSBs in HCC. The depletion of TKT effectively mitigates the radioresistance of HCC, both in vitro and in mouse xenograft models. Moreover, high TKT expression confers resistance of RT in clinical HCC patients, establishing TKT as a marker for assessing the response of HCC patients who received cancer RT. In summary, our findings reveal a novel mechanism by which TKT contributes to the radioresistance of HCC. Overall, we identify the TKT-PARP1 axis as a promising potential therapeutic target for improving RT outcomes in HCC.

## Introduction

Liver cancer currently ranks as the fifth most prevalent primary malignancy and the second leading cause of cancer-related mortality on a global scale [1]. In 2020, there were over 900,000 new cases of liver cancer and approximately 830,000 liver cancer-related deaths worldwide. Notably, 45.3% of the world’s liver cancer cases and 47.1% of its associated deaths occurred in China [2]. The incidence and mortality rates for liver cancer are anticipated to increase, underscoring its status as a significant global health concern. Hepatocellular carcinoma (HCC) accounts for approximately 80-90% of all primary liver cancer cases [3]. Despite hepatic resection and liver transplantation being primary treatments for HCC, the 5-year survival rate remains less than 20%, primarily due to the high recurrence rate post-procedural. This underscores the pressing need to identify novel HCC treatments.

Owing to the resistance to chemotherapy and the low survival rates following radical surgery, DNA damage-based radiotherapy (RT) assumes a crucial role in the treatment of patients with metastatic HCC and unresectable HCC [4–7]. RT induces extensive base damage, single-strand breaks (SSBs), and double-strand breaks (DSBs) by generating radiolysis radicals that target the sugar-phosphate backbone in intracellular DNA [8]. Among various forms of DNA damage, DSBs are the most harmful and prevalent form, as unrepaired or improperly repaired DSBs can lead to substantial chromosomal alterations, including deletions, insertions, inversions, translocations, and genomic rearrangements [9–11]. Ultimately, this can trigger genomic instability, apoptosis, senescence, or tumorigenesis. DSBs can be mended through two major, distinct pathways: homologous recombination (HR) and non-homologous end joining (NHEJ). These pathways vary in terms of repair accuracy and the necessity for a homologous template. NHEJ is a swift and non-specific ligation process that joins the broken DNA ends, often exhibits with little or no homology. In contrast, HR is a slower, higher-fidelity pathway that relies on homologous sequences from a sister chromatid or homologous chromosome as a template for repair [12, 13]. While many patients with metastatic [14] or unresectable HCC [15] have benefitted from RT [7], radioresistance stands out as a primary factor contributing to RT failures in HCC patients [16], significantly impacting HCC prognosis. Consequently, the exploration of new targets that could potentially enhance radiosensitivity in HCC patients is of great interest.

Transketolase (TKT) assumes a pivotal role as a key rate-limiting enzyme within the non-oxidative branches of the pentose phosphate pathway (PPP). This pathway is responsible for more than 85% generation of ribose-5-phosphate (R5P), a vital substrate essential for DNA and RNA biosynthesis [17–19]. TKT facilitates a series of reversible reactions within the non-oxidative PPP, enabling the conversion of R5P and xylulose-5-phosphate (Xu5P) into glyceraldehyde-3-phosphate (G3P) and sedoheptulose-7-phosphate (S7P) [20]. Furthermore, it mediates the conversion of Xu5P and erythrose-4-phosphate (E4P) into fructose-6-phosphate (F6P) and G3P [20]. Prior research has demonstrated TKT overexpression in HCC and breast cancer, was associated with metastatic potential and poor prognosis [19, 21, 22]. Notably, malonylation of TKT impacts its enzymatic activity, resulting in reduced R5P production and the onset of DNA damage in colorectal cancer [23].

Poly (ADP-ribose) polymerase 1 (PARP1) is a well-studied member of the PARP superfamily. Moreover, PARP1 is ubiquitous and abundant, participating in a range of cellular processes, including DNA repair and transcription [24–26]. Upon DNA damage, PARP1 activates itself through autoPARylation, subsequently PARylating numerous other acceptor proteins using nicotinamide adenine dinucleotide (NAD+) as a donor [27, 28]. This post-translational modification, known as PARylation, not only regulates protein function but also recruits additional proteins to damaged sites for aiding of DNA repair [29].

A previous study indicated that TKT deficiency in hepatocytes reduced liver DNA damage by elevating R5P and nucleotide levels [17]. TKT depletion suppressed cell proliferation by inducing R5P accumulation in HCC cells [21], highlighting distinct roles for TKT in normal cells and HCC cells. In the current study, we explored the functional role of TKT in the repair of double-strand breaks (DSBs) in HCC. TKT exerts a stimulatory effect on both homologous recombination (HR) and non-homologous end joining (NHEJ) repair pathways by interacting with PARP1. Mechanistically, PARP1 can PARylate TKT, and in turn, TKT promotes the autoPARylation of PARP1 in response to DSBs in HCC. Consequently, TKT confers radioresistance to HCC, both in vitro and in mouse xenograft models. High TKT expression leads to radioresistance in clinical HCC patients. Our study indicates that TKT-PARP1 axis as a promising therapeutic target for enhancing RT outcomes in HCC.

## Results

### TKT is involved in DSB repair of HCC

TKT expression exhibited a marked upregulation in HCC tissues when compared to peri-tumoral tissues (Supplementary Fig. S1A–C). The expression of TKT showed a significant increase in tandem with the progression of HCC tumor grade (Supplementary Fig. S1D), and high TKT expression was indicative of a poor prognosis for HCC, as per TCGA dataset (Supplementary Fig. S1E).

**Fig. 1:**
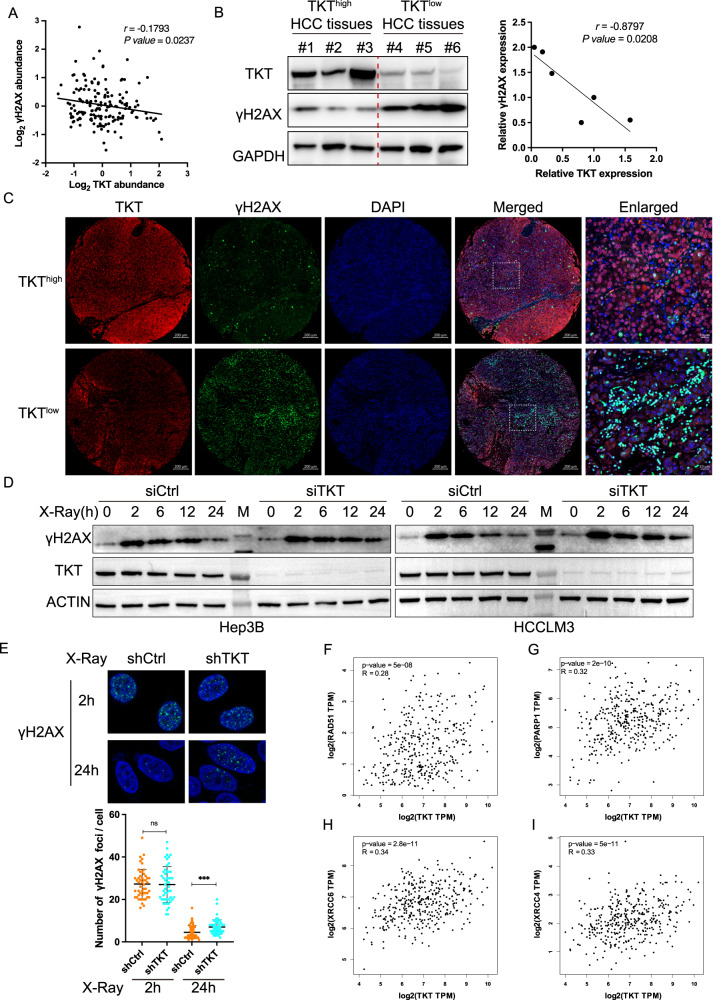
TKT is involved in DSB repair in HCC. **A** The correlation analysis between TKT and γH2AX expression using the published proteomic and phosphoproteomics dataset of 159 HCC patients from Fudan University. **B** The expression levels of γH2AX in TKT high and low tissues from 6 patients with HCC were compared using WB. **C** Representative MIF staining micrographs showing TKT (low or high) and γH2AX expression in an HCC microarray. Scale bar, 200 μm (TKT, γH2AX, DAPI and Merged) and 20 μm (Enlarged). **D** The Hep3B (Left) and HCCLM3 (Right) cells were collected without treatment, or at 2 h, 6 h, 12 h and 24 h post 4 Gy X-Ray treatment with siCtrl or siTKT transfection. Cell lysates were immunoblotted with the indicated antibodies. M, protein ladder. **E** Representative fluorescence images (green, γH2AX; blue, DAPI) and quantification of γH2AX immunostaining in cells at the indicated time after X-Ray treatment with or without TKT depletion. The correlation analysis between TKT and RAD51 (**F**), PARP1 (**G**), XRCC6 (**H**), XRCC4 (**I**) expression in HCC tissues by Spearman correlation analysis from TCGA database. Adapted from GEPIA: http://gepia.cancer-pku.cn/. TPM, transcripts per million.

To delve into the potential involvement of TKT in DSB repairment of HCC, we initially investigated the correlation between DSB levels (phosphorylated form of the histone variant H2AX, γH2AX) and TKT expression. This analysis was based on a previously published proteomic and phosphoproteomic dataset comprising 159 HCC patients [30]. The findings revealed a negative correlation between TKT expression and γH2AX in these 159 HCC tissues (*r* = −0.1793, *p* = 0.0237, Fig. 1A), suggesting a pivotal regulatory role for TKT in HCC’s DSB repair. We corroborated this correlation through western blotting (WB) in six HCC tissues. The WB results demonstrated that HCC tissues with elevated TKT protein levels displayed reduced γH2AX signals, while the reverse conditions were observed in HCC tissues with low TKT protein levels (Fig. 1B). Furthermore, this correlation was affirmed using multiplex immunofluorescence (MIF) staining of TKT and γH2AX in an HCC tissue microarray. The MIF results validated that HCC samples with a high TKT expression exhibited significantly lower γH2AX expression (Fig. 1C), underscoring the close association between high TKT expression and diminished levels of DSBs. These findings underscored the strong link between high TKT expression and reduced DSBs in HCC.

We extended our investigation to ascertain whether TKT regulates DSB repair in HCC cells by scrutinizing the kinetics of γH2AX induction following a 4 Gy X-Ray treatment in two HCC cell lines (Hep3B and HCCLM3) transfected with negative control siRNA (siCtrl) or siRNA against TKT (siTKT). The WB results unveiled that the γH2AX signal rapidly diminished post X-Ray treatment in control HCC cells (within 12 h), whereas it persisted for up to 24 h in TKT knockdown cells of Hep3B and HCCLM3 (Fig. 1D). This suggested that TKT-depleted HCC cells exhibited impaired DSB repair efficiency. Moreover, the γH2AX foci in TKT knockdown HCCLM3 cells were significantly higher than those in Ctrl HCCLM3 cells 24 h after X-Ray treatment (Fig. 1E). However, no significant difference was observed between shCtrl and shTKT cells at 2 h post X-Ray. These outcomes indicated that TKT knockdown did not impact the recruitment of γH2AX but did affect the clearance of γH2AX post DNA damage. Spearman correlation analysis using the TCGA database illustrated that TKT expression was positively associated with factors involved in the HR or NHEJ pathway (including RAD51, PARP1, XRCC6, and XRCC4) within HCC tissues (Fig. 1F–I). In summary, these findings strongly suggest that high TKT expression is indicative of fewer DSBs and that TKT may play a stimulatory role in DSB repair in HCC.

### TKT depletion suppresses NHEJ and HR repair in response to DSBs

**Fig. 2:**
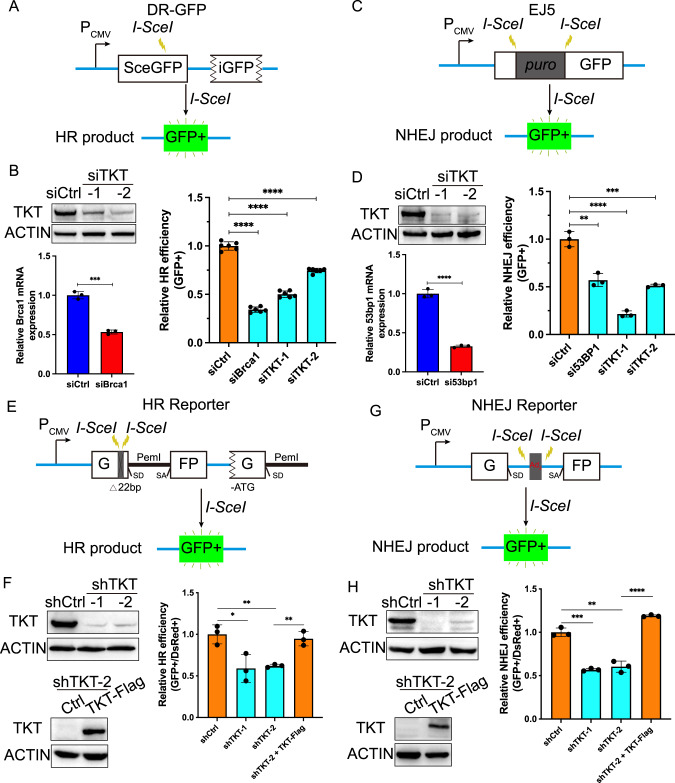
TKT depletion suppresses DSB repair in HCC. Schematic of the DR-GFP (**A**) and EJ5-GFP (**C**) reporter systems and relative HR (**B**) and NHEJ (**D**) repair efficiency in U2OS cells after being transfected the indicated siRNAs. Immunoblots of TKT and quantitative PCR (qPCR) analysis of BRCA1 (**B**) or 53BP1 (**D**) in U2OS cells after being transfected with the indicated siRNAs. ACTIN is used as a loading control. siBrca1 and si53bp1 are used as a positive control. Statistical analyses are presented as the mean±s.d. from three independent experiments. **p < 0.01; ***p < 0.001; ****p < 0.0001 of Student’s *t* test. Schematic of the HR (**E**) and NHEJ (**G**) reporter systems and relative HR (**F**) and NHEJ (**H**) repair efficiency in HCCLM3 cells with TKT stable knockdown. Immunoblots of TKT (**F**–**H**) in HCCLM3-shCtrl or shTKT cells. ACTIN is used as a loading control. Statistical analyses are presented as the mean±s.d. from three independent experiments. *p < 0.05; **p < 0.01; ***p < 0.001; ****p < 0.0001 of Student’s *t* test.

To investigate whether TKT influences the HR or NHEJ repair pathways, we employed two well-established U2OS cell lines, each harboring a chromosomally integrated reporter cassette (DR-GFP or EJ5-GFP), which facilitates the quantitative assessment of HR or NHEJ-mediated repair events [13, 16, 31]. TKT was downregulated using siTKT in DR-GFP and EJ5-GFP U2OS reporter cell lines, and the repair efficiency of HR and NHEJ was separately measured by flow cytometry (FACS). The results unequivocally demonstrated that TKT depletion significantly hindered the repair efficiency of both HR and NHEJ (Fig. 2A–D, Supplementary Fig. S2A–H).

To further validate TKT’s regulatory role in HCC, we transfected additional well-established linearized HR or NHEJ reporter constructs through in vitro I-SceI digestion into HCCLM3 cells, together with a DsRed plasmid for normalizing the transfection efficiency [32, 33]. Using extrachromosomal assays, we assessed the repair efficiency of HR and NHEJ in TKT-depleted HCCLM3 cells. The findings revealed that both HR and NHEJ repair were markedly suppressed by TKT knockdown in HCCLM3 cells (Fig. 2E–H, Supplementary Fig. S2I–P). We also found that TKT could significantly rescue the decline of HR and NHEJ efficiency, which was caused by TKT knockdown in HCCLM3 cells (Fig. 2F, H and Supplementary Fig. S2I–P), further affirming TKT’s stimulatory effect in HCC.

The regulatory role of TKT in DSB repair within HCC was consistent with the outcomes obtained from the DR-GFP and EJ5-GFP assays. Subsequently, we depleted TKT in Hep3B cells which containing a novel chromosomally integrated HR-NHEJ double reporter cassette, allowing simultaneous detection of HR and NHEJ at the same chromosomal site [9]. In concordance with the TKT depletion experiments in DR-GFP and EJ5-GFP U2OS cells, as well as the extrachromosomal assays in HCCLM3 cells, it was observed that TKT depletion significantly hampered both HR and NHEJ in Hep3B cells housing the HR-NHEJ double reporter cassette (Fig. S2M).Collectively, these experiments provide compelling evidence that TKT depletion hinders both HR and NHEJ repair mechanisms in response to DSBs.

### DNA damage increases TKT-PARP1 interactions

Previous research has hinted at the potential interaction between TKT and PARP1 in neonatal rat cardiomyocytes subjected to oxygen–glucose deprivation [34], a condition known to induce substantial DNA damage, including DSBs. PARP1, known as an early response factor to DNA damage, can be activated through autoPARylation in response to both SSBs and DSBs. In our prior studies, we observed the persistence of the TKT-PARP1 interaction in HCC tumor samples [35] and X-Ray treated 293FT cells through the results of immunoprecipitation (IP) experiments coupled with mass spectrometry (MS) [9] (Supplementary Fig. S3A, B).

**Fig. 3:**
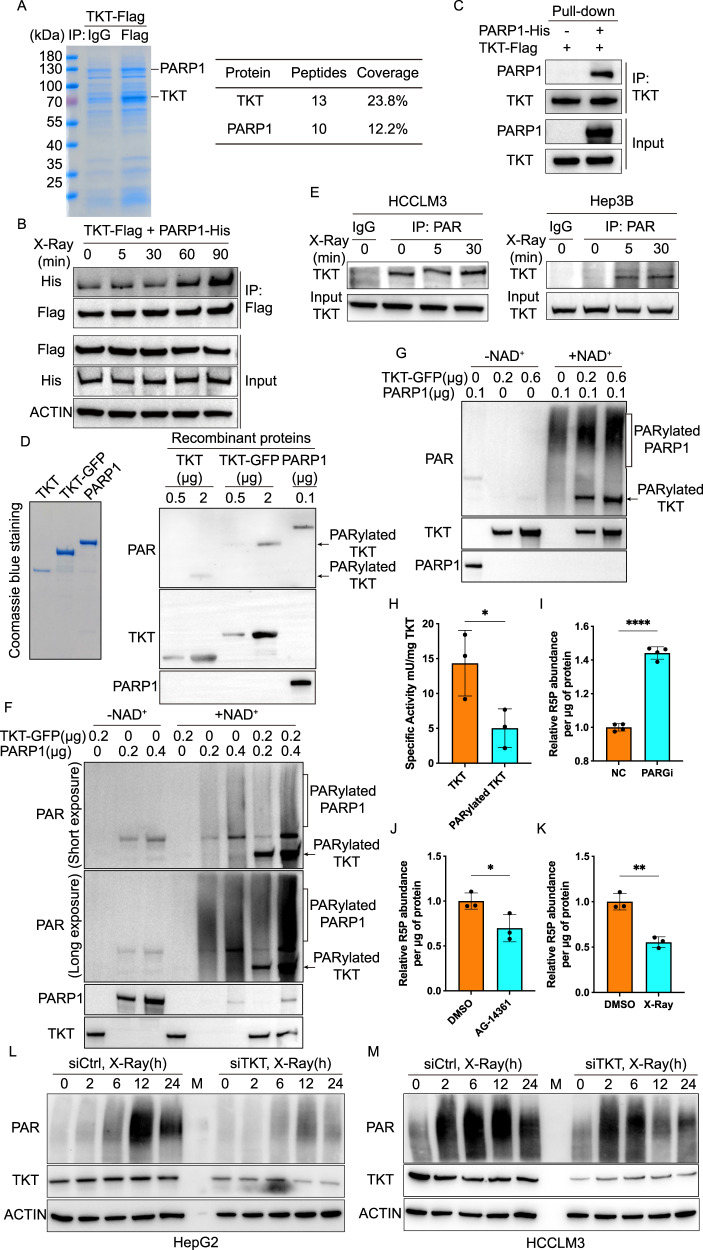
DNA damage increases TKT-PARP1 interaction and PARP1 directly PARylates TKT in HCC. **A** Coomassie staining of the TKT complex separated by SDS–PAGE. Hep3B cells stably expressing TKT-Flag were used for immunoprecipitation with an anti-Flag or anti-IgG antibody. TKT and TKT-interacting proteins, including PARP1, are indicated. The gel pieces containing regions of interest were analyzed by mass spectrometry. The list shows the peptides and coverage of TKT and PARP1 from the mass spectrometry. **B** Co-IP and WB analysis of the interaction between TKT and PARP1 in TKT-Flag and PARP1-His co-transfected Hep3B cells at different time points after X-Ray treatment. **C** Immunoblotting of indicated proteins in the in vitro pull-down assay of PARP1-His and TKT-Flag. **D** Coomassie staining of recombinant TKT, TKT-GFP and PARP1 protein (Left) and immunoblotting detection of PARylated TKT and PARP1 with the recombinant TKT, TKT-GFP and PARP1 protein (Right). **E** Co-IP and WB analysis of PARylated TKT in HCCLM3 and Hep3B cells in response to X-Ray. **F**, **G** Immunoblotting detection of PARylated TKT and PARP1 with or without PARP1 in an in vitro PARylation assay. **H** The activity detection of TKT and PARylated TKT by Transketolase Activity Assay Kit. Statistical analyses are presented as the mean ± s.d. from three independent experiments. *p < 0.05 of Student’s *t* test. The R5P abundance in DMSO or PARG inhibitor (**I**), PARP1 inhibitor (**J**), X-Ray (**K**) treated Hep3B cells. Statistical analyses are presented as the mean±s.d. from three independent experiments. *p < 0.05; **p < 0.01; ****p < 0.0001 of Student’s *t* test. **L**, **M** Immunoblotting detection of PARylation in siCtrl or siTKT transfected HepG2 and HCCLM3 cells at different time points after X-Ray treatment.

To verify the existence of an interaction between TKT and PARP1, we overexpressed TKT-Flag in Hep3B cells and conducted an IP-MS analysis using the Flag tag. The IP-MS results unequivocally identified PARP1 as a binding protein of TKT in HCC cells (Fig. 3A). To substantiate the interaction between TKT and PARP1 in HCC cells and determine whether it relied on DNA damage, we performed a Co-IP assay in Hep3B cells. In this assay, PARP1-Flag plasmid or TKT-Flag plasmid was individually transfected. Whole-cell lysates from Hep3B cells collected before treatment and 5, 30, 60 min following an 8 Gy X-Ray treatment were prepared and subjected to IP assays employing an anti-Flag antibody. In agreement with the MS data, immunoblotting analysis demonstrated an augmentation of the TKT-PARP1 interaction in the immunoprecipitates of TKT or PARP1 at the 60-min sample after the 8 Gy X-Ray treatment in Hep3B cells (Supplementary Fig. S3C, D).

Furthermore, we detected the interaction between TKT and PARP1 in PARP1-His and TKT-Flag co-transfected Hep3B cells (Fig. 3B). Additionally, in vitro pull-down assays provided further confirmation of the direct interaction between TKT and PARP1 (Fig. 3C), establishing that TKT indeed interacts directly with PARP1. To map the interaction domain of TKT with PARP1, we transfected TKT or PARP1 truncation mutants [36] with PARP1 or TKT full-length plasmid into Hep3B cells respectively. Co-IP experiments showed that the N, M domains of TKT and the ZnF, BRCT, and WGR domains of PARP1 mediated the interaction between TKT and PARP1 in HCC cells (Supplementary Fig. S3E, F).

### TKT undergoes PARylation by PARP1 In Vivo and In Vitro

Given that TKT demonstrated a direct interaction with PARP1 in a DNA damage-dependent fashion, we sought to determine whether TKT could be directly PARylated by PARP1. Initially, we purified recombinant TKT and GFP-tagged TKT (GFP-TKT) protein from HEK293F cells and subjected them to immunoblotting using a PAR-specific antibody. The WB results clearly revealed that PARylated TKT was detectable, and its intensity increased with rising concentrations of TKT/GFP-TKT protein (Fig. 3D), confirming the PARylation of TKT.

Subsequently, we investigated whether TKT could undergo PARylation in vivo and whether this PARylation was contingent on DNA damage within HCC cells. We immunoprecipitated PARylated proteins using Af1521 Macrodomain (PAR/MAR) Affinity Resins from HCCLM3 cells and Hep3B cells treated with 8 Gy X-Ray, followed by immunoblotting analysis. The WB results indicated an increase in TKT PARylation in response to DNA damage in HCC cells (Fig. 3E). To further substantiate that PARP1 directly mediated the PARylation of TKT, we incubated increasing amounts of recombinant GFP-TKT protein with PARP1 or increasing amounts of PARP1 with GFP-TKT. Subsequent immunoblotting with a PAR-specific antibody in an in vitro assay clearly showed an augmentation in the signal of the PARylated TKT band upon the addition of PARP1 (Fig. 3F, G).

To assess the impact of PARylation on TKT’s activity, we utilized a Transketolase Activity Assay Kit. The results underscored that PARylation significantly inhibited TKT’s activity (Fig. 3H). Furthermore, we evaluated R5P abundance in cells treated with X-Ray, or a PARG inhibitor, a compound known to sustain the PARylation level of TKT, and a PARP1 inhibitor (AG-14361). The findings indicated that PARG inhibitor and X-Ray treatment substantially increased R5P level (Fig. 3I, K), and PARP1 inhibitor treatment significantly decreased R5P level in HCC cells (Fig. 3J). These data demonstrated the PARylation of TKT could increase the level of R5P in HCC cells.

Intriguingly, beyond the enhanced TKT PARylation, we unexpectedly discovered that TKT had the capacity to enhance the auto-PARylation of PARP1 (Fig. 3F, G). To verify whether TKT indeed boosted the auto-PARylation of PARP1, we examined the PAR signal in TKT-depleted HepG2, HCCLM3, Hep3B, and Huh7 cells. The results revealed a significant reduction in the auto-PARylation of PARP1 in TKT-depleted HCC cells compared to control cells in response to X-Ray treatment (Fig. 3L, M, Supplementary Fig. S3G, H). These results collectively suggest that DNA damage elevates the interaction between TKT and PARP1, and PARP1 can PARylate TKT in a DNA damage-dependent manner. The PARylation of TKT hampers its activity while increasing R5P synthesis, and concurrently, TKT significantly enhances the auto-PARylation of PARP1 in response to DNA damage.

### TKT increases the radioresistance of HCC

**Fig. 4:**
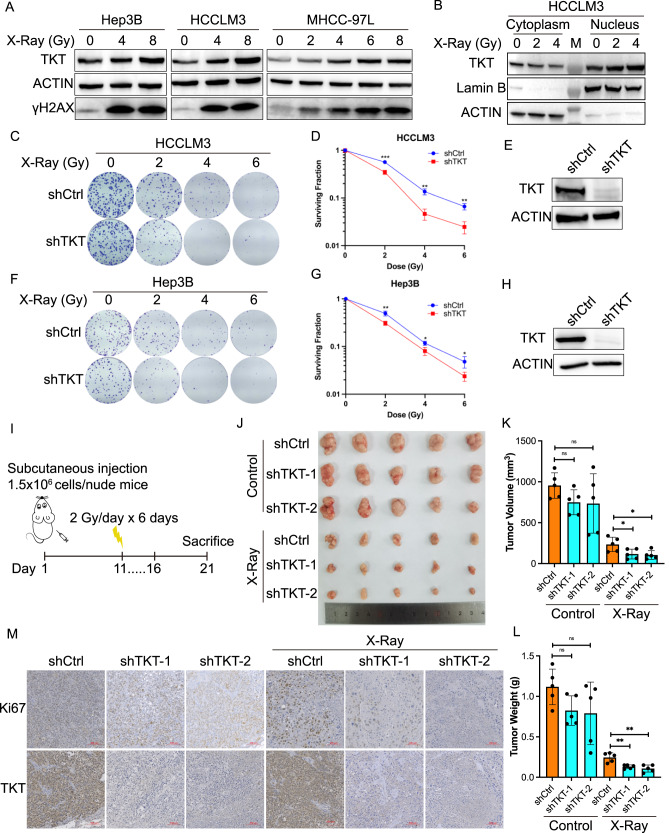
TKT induces radioresistance of HCC in vitro and in vivo. **A** Immunoblotting analysis of TKT expression in Hep3B, HCCLM3, and MHCC-97L cells at 8 h after different doses of X-Ray treatment. **B** Immunoblotting analysis of TKT expression in nucleus and cytoplasm of HCCLM3 cells at 8 h after different doses of X-Ray treatment. Lamin B and ACTIN are used as internal control of nucleus and cytoplasm. Colony formation assays are applied to assess the survival of HCCLM3 (**C**–**E**) and Hep3B (**F**–**H**) cells treated with different doses of X-Ray with stable TKT knockdown and Immunoblotting analysis of TKT in TKT stably knockdown HCCLM3 (**E**) and Hep3B (**H**) cells. Representative images (**C**, **F**); quantified cell surviving curves (**D**, **G**) are presented as the mean±s.d. of three independent experiments. ***p < 0.001; **p < 0.01; *p < 0.05. **I** Schematic illustration of the radiotherapy schemes in the xenograft mouse models. Tumor image (**J**), volume (**K**) and weight (**L**) of tumors harvested in nude mice subcutaneously injected with TKT-knockdown Hep3B cells and its control, with/without X-Ray. *n* = 5; n.s. no significance. **p < 0.01; *p < 0.05 of Student’s *t* test. **M** Representative immunohistochemistry images of the expressions of TKT and Ki67 in the xenograft tumors in each group. Scale bar, 100 μm.

A prior investigation had indicated an upregulation of TKT mRNA expression in radioresistant glioblastoma cells [37] (Supplementary Fig. S4A). We thus sought to determine if TKT could engender radioresistance in HCC. To address this, we initiated our exploration by assessing the protein-level expression of TKT in response to X-Ray treatment in three HCC cell lines. The outcomes disclosed a notable increase in TKT expression in Hep3B, HCCLM3, and MHCC-97L cells following exposure to X-Ray, and this upsurge in TKT exhibited a dose-dependent relationship with the X-Ray dosage (Fig. 4A).

To delve deeper into whether the heightened TKT expression was prevalent in the nucleus or cytoplasm, we performed a cytoplasmic and nuclear fractionation assay on HCCLM3 cells subjected to X-Ray treatment. The WB results revealed the presence of TKT in both the nucleus and cytoplasm, with DNA damage predominantly triggering an increase in nuclear TKT (Fig. 4B).

For a more comprehensive examination of TKT’s role in radioresistance in HCC, we employed shRNA to stably suppress TKT expression in two HCC cell lines. Subsequently, we conducted a clonogenic assay to assess the colony-forming ability after X-Ray treatment. The results from the colony formation assay unequivocally demonstrated that TKT knockdown significantly reduced the number of colonies in HCCLM3 (Fig. 4C–E) and Hep3B (Fig. 4F–H) cells following X-Ray treatment, in comparison to the respective shCtrl cells. This compelling evidence indicated that TKT indeed conferred radioresistance to HCC cells.

To further explore whether TKT could bolster radioresistance in an in vivo HCC model, we established a subcutaneous xenograft tumor model. Nude mice were injected subcutaneously with either shCtrl or shTKT-1/2 Hep3B cells. After the tumors grown and reached a volume of approximately 100 mm^3^, the nude mice received either no treatment or irradiation. The irradiation consisted of a daily fractionated dose of 2 Gy of X-Ray for six consecutive days (Fig. 4I). The results demonstrated no significant difference in tumor volume and weight between the unirradiated shCtrl group and the shTKT-1/2 groups. However, post-irradiation, the tumors in the shTKT-1/2 groups were significantly smaller and lighter than those in the shCtrl group, suggesting that the TKT-depleted tumors exhibited a considerably heightened radiosensitivity (Fig. 4J–M). Collectively, these findings provided robust evidence that TKT could induce radioresistance in HCC, both in vitro and in vivo.

### TKT predicts RT vulnerability of HCC

**Fig. 5:**
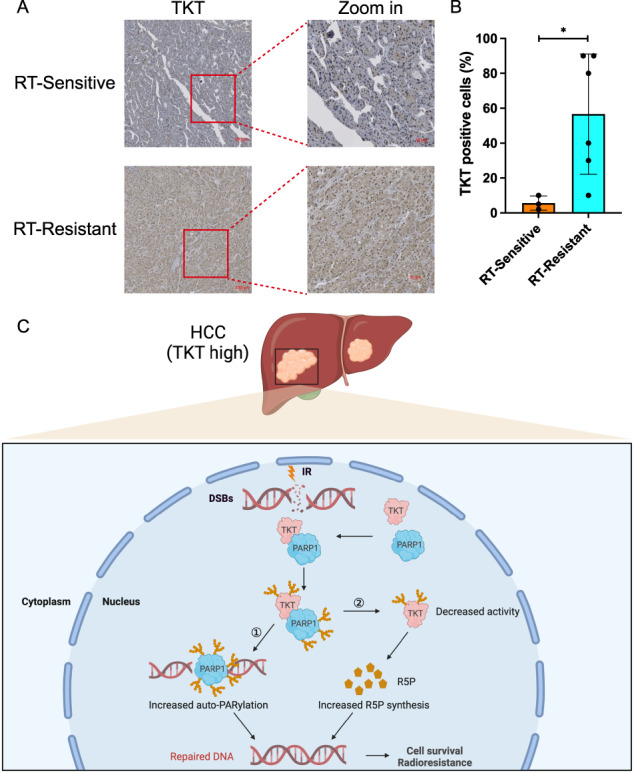
TKT confers radioresistance in HCC clinical samples. **A** Representative immunohistochemistry images of the expressions of TKT in RT-Sensitive and RT-resistant HCC samples. Scale bar, 100 μm (Left) and 50 μm (Right). **B** Statistical analyses of the expression level of TKT from all HCC immunohistochemistry staining. *p < 0.05 of Student’s *t* test. **C** Schematic diagram summarizing TKT induces radioresistance via promoting DSB repair in HCC (Created by BioRender.com).

To ascertain whether TKT could serve as a predictor of radioresistance in clinical HCC patients, we procured 9 samples from HCC patients who had undergone RT. Among these 9 patients, 3 experienced a reduction in tumor size, while 6 displayed no discernible change following RT. Subsequently, we conducted immunohistochemistry (IHC) staining to evaluate TKT expression in these 9 HCC samples. The IHC results unequivocally indicated that high TKT expression corresponded to radioresistance, whereas low TKT expression correlated with radiosensitivity in HCC patients (Fig. 5A, B).

In light of these findings, we deduced that TKT bestows radioresistance by interacting with PARP1, thereby facilitating the auto-PARylation of PARP1, increasing R5P synthesis, and ultimately enhancing the proficiency of DSB repair in HCC (Fig. 5C). In summation, these results underscored the strong association between high TKT expression in HCC and reduced RT effectiveness, resulting in poorer survival rates for HCC patients. As such, TKT expression can be regarded as a valuable marker for assessing the response of cancer treated with RT in the context of HCC.

## Discussion

RT stands as one of the principal modalities for managing patients with metastatic and unresectable HCC. Despite substantial advancements in the efficacy of RT, the clinical challenge of radioresistance in HCC persists. The underlying mechanism contributing to the development of radioresistance in HCC necessitates further exploration. Transketolase (TKT), a pivotal rate-limiting enzyme in the non-oxidative pentose phosphate pathway (PPP), plays a crucial role in supplying over 85% of ribose-5-phosphate (R5P) required for DNA and RNA biosynthesis. RT inflicts substantial DNA DSBs in cells, demanding a substantial supply of nucleotides for the survival of tumor cells.

Significantly, previous studies have presented a dual role of TKT in normal hepatocytes and HCC cells. TKT depletion in hepatocytes has been shown to reduce liver DNA damage induced by diethylnitrosamine (DEN), as it elevates R5P and nucleotide levels [17]. Conversely, in HCC cells, TKT depletion has been observed to hinder cell proliferation by inducing R5P accumulation [21]. The upregulation of TKT in HCC [19, 21], coupled with its association with poor prognosis, suggests that TKT could serve as a novel therapeutic target for HCC.

In our study, we analyzed the correlation between TKT and γH2AX, a marker of DSBs, using proteomic and phosphoproteomic datasets from 159 HCC patients. This analysis revealed a significant negative correlation between TKT and γH2AX (*r* = −0.1793, *p* = 0.0237). We further corroborated this negative correlation through WB analysis of six HCC samples and multiplex immunofluorescence staining of an HCC tissue microarray. Our findings indicate that TKT-depleted HCC cells exhibit a significantly reduced capacity for DSB repair, suggesting that TKT depletion impedes DSB repair in HCC.

DSBs can be repaired through two pathways: precise HR and error-prone NHEJ. Using the well-established DR-GFP and EJ5 U2OS cell lines, we observed that TKT depletion significantly hindered both HR and NHEJ repair. This inhibitory role of TKT depletion in HCC cells was further confirmed, one utilizing a novel HR-NHEJ double reporter cassette chromosomally integrated [9] and the other employing the extrachromosomal assay of linearized HR or NHEJ reporters [33].

The interaction between PARP1 and TKT was examined in primary neonatal rat cardiomyocytes under conditions of oxygen–glucose deprivation [34], known to induce extensive DSBs in cells [38]. In HCC cells, the TKT-PARP1 interaction was observed to increase in response to DSBs, a phenomenon confirmed through in vitro assays. Furthermore, the study observed robust PARylation of TKT protein upon exposure to recombinant TKT proteins, where the PARylation of TKT was notably enhanced by PARP1. The PARylation of TKT was also detected in HCCLM3 and Hep3B cells following X-Ray treatment. Remarkably, this PARylation substantially inhibited TKT activity and increased R5P synthesis in HCC cells.

Interestingly, the study unveiled that in response to DNA damage in HCC cells, TKT could augment the auto-PARylation of PARP1 both in vitro and in vivo. However, the precise PARylation sites on TKT and the mechanisms by which TKT promotes the auto-PARylation of PARP1 remain subjects for future investigation.

A previous study highlighted the upregulation of TKT mRNA in radioresistant glioblastoma cells [37]. In our investigation, we also observed a marked increase in TKT expression in HCC cells following X-Ray treatment, primarily in the nuclear compartment. Given TKT’s potential role in mediating radioresistance in HCC, we subjected TKT-depleted HCC cells to varying doses of X-Ray and assessed the impact of TKT depletion on cell survival. Our findings revealed that TKT depletion significantly enhanced the radiosensitivity of HCC cells, both in vitro and in vivo. Consistent with previous studies that showed TKT knockdown suppressed tumor growth [19, 21], our mouse xenograft models displayed a reduction in tumor weight and size, although not reaching statistical significance. With TKT conferring radioresistance in both HCC cells and mouse xenograft models, we further examined TKT expression in clinical HCC samples from patients who had undergone RT. This analysis involving 9 clinical HCC samples, and we identified significantly higher TKT expression in radioresistant HCC samples compared to radiosensitive HCC samples. Therefore, our study suggests that TKT expression could serve as a promising marker for predicting the vulnerability of HCC to RT.

In summary, this study underscores the potential of TKT depletion to overcome radioresistance in HCC and provides insights into a novel mechanism of HCC radioresistance through the TKT-PARP1 axis-mediated DNA double-strand break (DSB) repair. Consequently, TKT inhibition emerges as a prospective strategy to enhance the sensitivity of HCC to RT.

## Materials and methods

### Clinical specimens

The samples of fresh tissue were obtained from Nanjing Drum Tower Hospital. All tissues were immediately put into liquid nitrogen after surgical resection. The specimens of HCC patients treated with radiotherapy were obtained between 2017 and 2018 from Nanjing Drum Tower Hospital, all of which were formalin-fixed paraffin-embedded (FFPE) samples. This study was performed in accordance with protocols approved by the Institutional Ethics Committee of Nanjing Drum Tower Hospital. All experimental procedures were complied with government policies and the guidelines of the Declaration of Helsinki. The analysis and experiments were conducted with the understanding and written consent of each participant.

### Cell culture

HCC cell lines (Hep3B, HCCLM3, Huh7 and MHCC-97L) were purchased from the cell bank of the Chinese Science Academy. HepG2 and HEK293T cells were obtained from the American Type Culture Collection. DR-GFP and EJ5 U2OS cells were a gift from Dr. Sijie Liu (Peking University, China). All these cells were cultured in DMEM (WISENT, China), containing 10% FBS with 1% penicillin streptomycin. HEK293F cells were cultured in SMM 293-TII medium (Sino Biological, China) in a shaker incubator at 120 rpm. All cells above were maintained at 37 °C and 5% CO2 in a humidified incubator.

### Plasmids and transfection

NHEJ and HR reporter plasmids were kindly provided by Dr. Zhiyong Mao (Tongji University, China). Full-length cDNA of human TKT was cloned into pCMV-Flag-puro vector or pCMV-GFP-Flag-puro vector. Full-length fragmented DNA encoding the PARP1 was cloned into the pCMV-His-HA or pCMV-Flag-His vector. Small-interfering RNAs (siRNAs) targeting TKT (#1, gccaucaucuauaacaacaau; #2, gcugagcugcugaagauguuu) were synthesized by GENERAL BIOL (China). The transfection of plasmid or siRNAs was performed using ExFect Transfection Reagent (Vazyme, T101-01) or PEI 40000 (YEASEN, 40816ES02) according to the instructions of the manufacturer for 24~48 h. Lentiviral vectors carrying a human TKT-specific shRNA were purchased from GENERAL BIOL. To knockdown of TKT, HCC cells were infected with lentiviruses. After infection, the infected cells were selected with puromycin (ThermoFisher, A1113803) for 2~3 days. The I-SceI lentivirus was packaged by the second-generation packaging system. In brief, 10 μg of the I-SceI vector, 10 μg of psPAX2 (Addgene, 12260) and 5 μg of pMD2.G (Addgene, 12259) were thoroughly mixed and co-transfected into HEK293T cells in 10 cm dish using PEI 40000 according to the manufacturer’s instructions. Forty-eight hours after transfection, the supernatant containing lentiviral particles was filtered and used to infect the indicated cells.

### Protein purification

The expression plasmid of TKT or PARP1 was transfected into HEK293F cells, respectively. When the cell density reached 2.0 ×10^6^ cells/ml, 1.5 mg of plasmid was pre-mixed with 4.5 mg of PEI 40000 for transiently transfected into one liter of HEK293F cell culture according to the manufacturer’s instructions. Cells were collected by 1000 g centrifugation after 72 h post-transfection and resuspended in binding buffer (50 mM Tris-HCl, pH 7.5, 150 mM NaCl, 0.01% NP-40, 10% glycerol, 1 mM PMSF, 2 mM TCEP) supplemented with 1x EDTA-free protease inhibitor cocktail (Beyotime, P1005). After sonication, lysates were clarified by ultracentrifugation at 40,000 rpm for 1 h at 4 °C. The supernatant containing recombinant proteins were incubated with anti-DYKDDDDK affinity beads (Smart-Lifesciences, SA042001) for 2 h on a rotator at 4 °C. After extensive wash with binding buffer supplemented with 2.5 mM MgCl_2_ and 5 mM ATP, target protein was eluted from beads by binding buffer plus 0.2 mg/ml Flag peptide (Sigma-Aldrich, F4799).

For the purification of PARP1-His protein, cells were resuspended in binding buffer supplemented with 20 mM imidazole. After ultracentrifugation, supernatant of protein lysate was combined with Ni-NTA agarose resin (YEASEN, 20502ES10) and eluted with 300 mM imidazole in binding buffer.

The protein-containing fractions were concentrated by ultrafiltration spin columns and further purified by size-exclusion column (GE Healthcare, Superose® 6 Increase 10/300 GL) in buffer containing 25 mM Tris (pH 8.0) and 150 mM NaCl. The peak fractions were collected for the subsequent analysis.

### Immunofluorescence

Cells were cultured on 35 mm Confocal Dishes and fixed with 4% paraformaldehyde (PFA) for 15 min at room temperature. Then the fixed cells were permeabilized with 0.25% Triton X-100 for 10 min and blocked with 1% goat serum for 1 h at room temperature, followed by overnight incubation with primary antibodies at 4 °C. Next day, the secondary antibody was added for 1 h incubation at room temperature. Samples were then washed three times and stained with DAPI for 2 min, followed by another three PBS washes. Images were collected on a scanning laser microscopy (Leica, USA).

### IP, mass spectrometry and pull-down assays

Cells transfected with the indicated expressing plasmids were collected and suspended in RIPA lysis buffer (Beyotime, P0013D) containing PMSF (Beyotime, ST506) and protease and phosphatase inhibitor cocktail (Beyotime, P1045) on ice for 30 min. After sonication, the cell lysate was collected by centrifugation at 13,000 g for 10 min at 4 °C. The supernatant was incubated with anti-Flag M2 magnetic beads (Sigma-Aldrich, M8823) for 2 h at 4 °C. After washed 5 times with TBST buffer, beads were mixed with 1× SDS sample loading buffer and boiled for 10 min before western blotting. Samples of TKT-Flag IP were resolved by SDS-PAGE and visualized by Coomassie blue staining. The band of interest was excised and subjected to a mass spectrometry analysis using LC-MS/MS.

Purified recombinant TKT and PARP1 proteins were pre-cleared with protein A/G beads (Beyotime, P2108) and incubated together for 2 h at 4 °C. Then, 2 μg of TKT antibody (Sino Biological, 206517-T36) was added to the mixture and rotated overnight at 4 °C. Next day, 25 μl of protein A/G beads were added and kept on a rotator at 4 °C for another 2 h. Finally, the pellets were intensively washed with TBST buffer and boiled for 10 min in 1× SDS sample buffer before western blotting.

### Western blotting

Cells or tissue homogenate were lysed with RIPA lysis buffer containing PMSF, protease and phosphatase inhibitor cocktail. The protein concentration of each sample was quantified by BCA protein quantification assay kit (Vazyme, E112-01). Twenty μg of lysate was separated using SDS-PAGE gels and transferred to PVDF membranes (Millipore). The membranes were blocked with 5% skim milk in TBST for 1 h at room temperature and probed with indicated primary antibody overnight at 4 °C. The next day, PVDF membranes were incubated with anti-mouse or anti-rabbit antibody for 1 h, after which the membranes were treated with ECL Chemiluminescence Kit (Vazyme, E423) and visualized in Tanon 4600 image system. Antibodies against β-actin (AC038), PARP1 (A0942), Lamin B (A16909), FLAG (AE092), γ-H2AX (Ser139) (AP0099), GAPDH (AC001), HIS (AE028) were from ABclonal, while antibody against PAR (ALX-804-220-R100) was from Enzo Life Sciences.

### HR and NHEJ repair assays

EJ5 U2OS and DR-GFP U2OS cell lines were employed to measure the efficiency of NHEJ and HR repair. EJ5 U2OS cells and DR-GFP U2OS cells grown in 6-well plates at a density of 1.5 × 10^5^ cells/well were pre-transfected with indicated siRNAs. At 24 h later, cells in each well were infected with 200 μl I-SceI lentivirus. Sixty hours later, cells were harvested and analyzed by flow cytometric analysis on a BD FACSAria III flow cytometer. 53bp1 and Brca1 were selected as positive control.

Linearized HR and NHEJ reporter systems were established to check repair efficiency in HCC cells. Briefly, the HCCLM3 cells were infected with lentivirus expressing control shRNA or shRNA targeting TKT to establish TKT-knockdown cells. The TKT-knockdown cells were seeded in 6-well plate. At 24 h later, cells in each well were transfected with 1.5 μg of NHEJ or 2 μg of HR which was linearized by in vitro I-SceI digestion, and 100 ng of DsRed for internal control. Cells were collected and analyzed by FACS at 48 h after transfection. The efficiency was calculated as the ratio of the percentage of GFP-positive to RFP-positive cells.

### Colony formation assays

Cells with TKT knockdown were seeded in 6-well plates at a density of 600 cells/well in triplicates. After 24 h, cells were respectively exposed to a total of 0, 2, 4 or 6 Gy by using an RS2000 X-Ray irradiator (Rad Source, RS2000pro-225). After 12 days of incubation, cells were fixed with 4% paraformaldehyde and stained with 0.5% crystal violet. Colonies containing more than 50 cells were counted.

### Immunohistochemical analysis and multiple immunofluorescence assays

Fresh tissue samples were fixed in 4% paraformaldehyde for 72 h and then dehydrated in alcohol with increasing concentrations of xylene. Next, the samples were embedded in paraffin, and the tissue wax blocks were sliced. Paraffin sections were deparaffinized and rehydrated after being incubated at 65 °C for 3 h. The slides were heated at 95 °C in 0.01 M citrate buffer (pH 6.0). The DAB kit (MXB Biotechnologies, KIT9720) was used according to the manufacturer’s protocol. After blocked, the tissues were incubated with a primary antibody at 4 °C overnight. The primary antibody was an anti-TKT antibody (ABclonal, A6314) or anti- Ki67 antibody (ABclonal, A20018). After washing three times with PBST, the sections were incubated with a secondary antibody for 1 h at room temperature. Hematoxylin counterstaining was completed, and all the sections were dehydrated and sealed.

Human HCC tissue microarray was purchased from chinaiwill (Live C-1401). A 4-Color Multiplex immunohistochemistry kit (PANOVUE, 10079100020) was used according to the manufacturer’s protocol. The primary antibody was an anti-TKT antibody or anti-γH2AX antibody (CST, 9718). The slides were imaged using a ZEISS Axioscan 7 microscope slide scanner.

### RNA extraction and RT-qPCR

Total RNA was extracted from cells using the FreeZol Reagent (Vazyme, R711-01) according to the manufacturer’s protocol, and quantified with a NanoDrop spectrophotometer. Approximately 1 μg of total RNA were used for reverse transcription using the HiScript III RT SuperMix (Vazyme, R323-01). RT-qPCR was performed with the ChamQ Universal SYBR qPCR Master Mix (Vazyme, Q711-02) on the Applied Biosystems QuantStudioTM 5 Real-Time PCR Instrument. Actin was used as an internal standard for normalization. The relative expression of target genes was calculated using the 2^−ΔΔCt^ method and plotted by GraphPad Prism 6. The primers were as follows: brca1-F:GAGGAACGGGCTTGGAAGAA; brca1-R: TGCATGGTATCCCTCTGCTG; 53bp1-F: GAAACGGCGCAGTAACGTC; 53bp1-R: TCCTGCCCCTACAGGTTTTACT; actin-F: TCACCATGGATGATGATATCGC; actin-R: ATAGGAATCCTTCTGACCCATGC.

### In vitro PARylation assay

The reactions were performed with recombinant PARP1 and TKT at 30 °C for 1 h in a reaction buffer (50 mM Tris-HCl, pH 7.5, 20 mM NaCl, 4 mM MgCl2 and 250 μM DTT) supplemented with 1 mM NAD^+^ and 100 ng activated DNA (Sigma-Aldrich, D4522). Reactions were stopped by adding SDS sample loading buffer and the samples were analyzed by Western blotting.

### Cellular PARylation assay

The Hep3B cells and HCCLM3 cells were treated with X-Ray and harvested at the indicated time points. Cells were lysed with RIPA lysis buffer on ice for 30 min. After sonication, the cell lysate was collected by centrifugation at 13,000 g for 10 min at 4 °C. The supernatant was incubated with Af1521 macrodomain (PAR/MAR) affinity resins (Tulip BioLabs, 2302) at 4 °C overnight. After washing 3 times, resins were mixed with 1× SDS sample loading buffer and boiled for 10 min before Western blotting.

### Animal experiments

Four-week-old BALB/c male nude mice were purchased from GemPharmatech. All mice were provided with food and water AD libitum and maintained in specific pathogen-free facilities on a 12 h light-dark cycle. All animal procedures were in compliance with the Guide for the Care and Use of Laboratory Animals. Mice were randomly assigned to subcutaneous injection with TKT knockdown or control Hep3B cells (1.5 × 10^6^/mouse). Eleven days after injection, radiation was given at a dose of 2 Gy for 6 consecutive days. Tumor volume (mm^3^) was calculated with formula: V = 0.5 × length × width^2^.

### Statistical analysis

All data were presented as means ± s.d. of at least three biological replicates samples. Statistical and significance analysis were performed using the GraphPad Prism 6 and regarded significant if *p* values were <0.05. Student’s *t* test was used for comparison of two different groups. Significant *p* values are indicated within the figures. *p* < 0.05 (*) is significant and *p* < 0.01 (**), *p* < 0.001 (***) and *p* < 0.0001 (****) are considered to be highly significant.

### Supplementary information

Supplemental material

## Data Availability

All data generated or analyzed during this study are included in this article and its supplementary information files.
