# Supplementary material for: TKT-PARP1 axis induces radioresistance by promoting DNA double-strand break repair in hepatocellular carcinoma
Source: Oncogene. 2024 Jan 12;43(9):682–92. doi: 10.1038/s41388-023-02935-9 (PMC10890932; doi:10.1038/s41388-023-02935-9)
Supplement: Supplementary file 1 — Supplemental material [file 41388_2023_2935_MOESM1_ESM.docx]

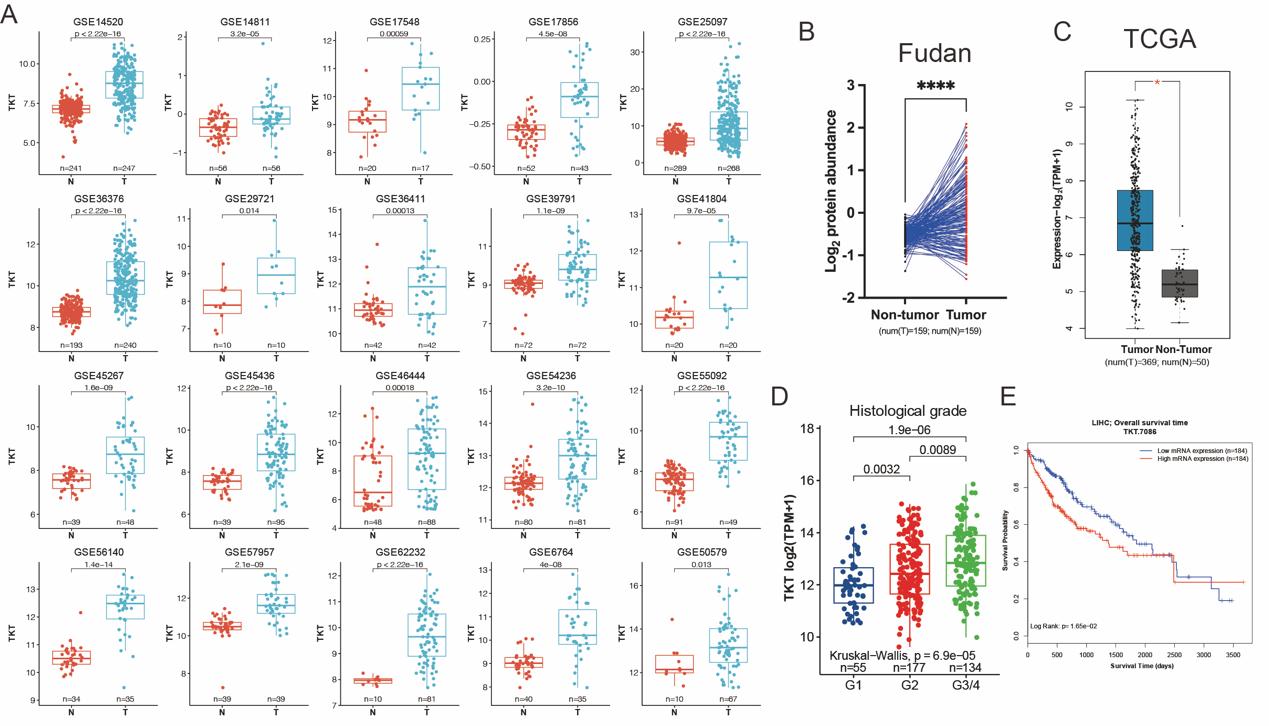


**Figure S1. TKT was upregulated in HCC and high expression of TKT predicted a poor prognosis of HCC.** (A-C) The expression pattern of TKT in HCC tumor tissues and non-tumor tissues based on GEO (A), Fudan data (B) and TCGA dataset (C). P values are indicated; ****p<0.0001; *p<0.05 of Student's t test. (D) TKT expression according to histologic grade of HCC in the TCGA dataset. P values are indicated. (E) High TKT expression levels predict worse OS based on the TCGA dataset. P values are indicated.


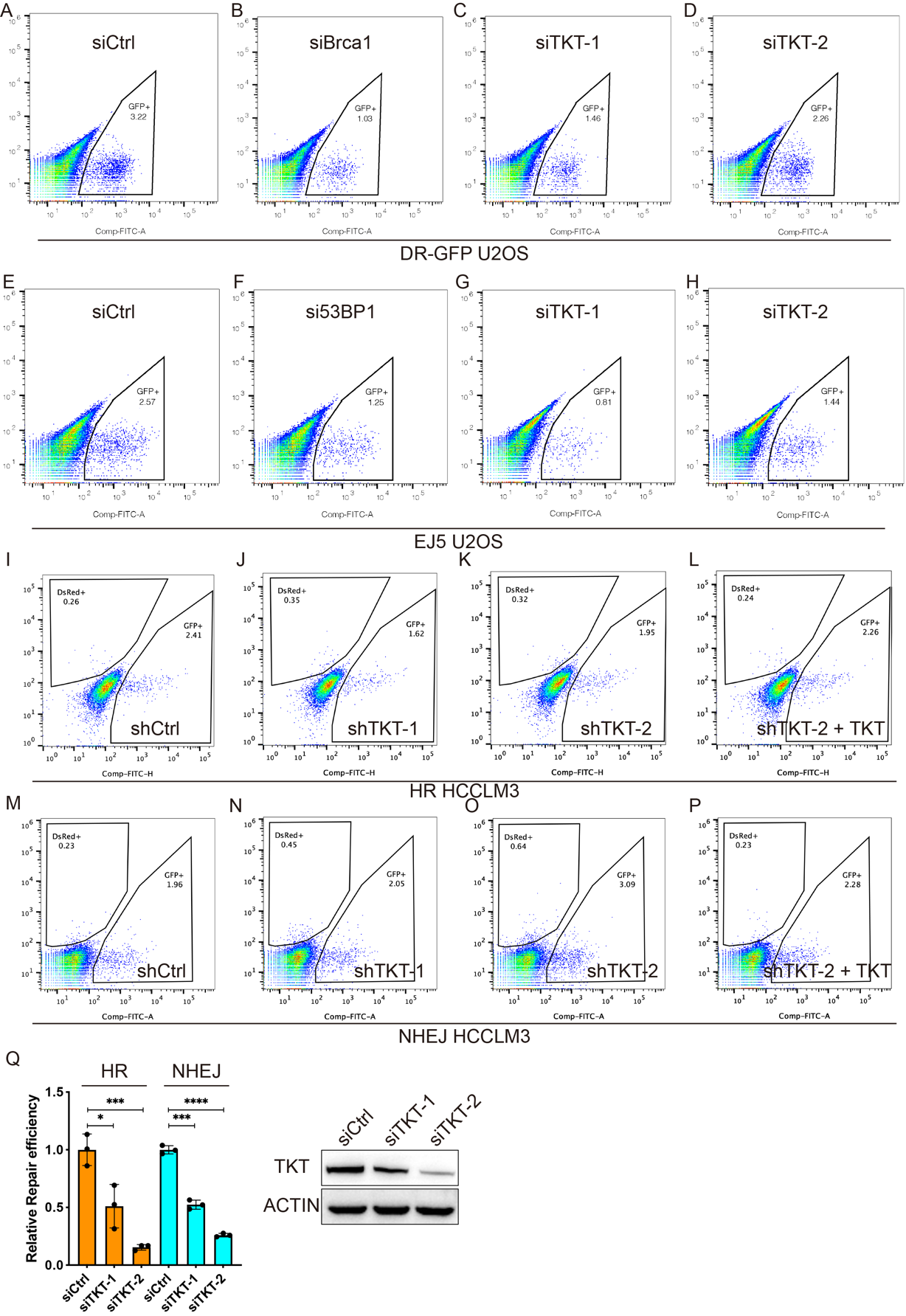


**Figure S2. TKT depletion inhibited both HR and NHEJ repair.** (A-H) Representative images of the raw FACS data in siCtrl, siBrca1, siTKT-1 or siTKT-2 transfected DR-GFP U2OS cells (A-D) or EJ5 U2OS cells (E-H). (I-P) The raw FACS data in linearized HR (I-L) or NHEJ (M-P) reporter transfected HCCLM3 cells. (Q) Relative HR and NHEJ repair efficiency in siCtrl or siTKT transfected Hep3B cells with chromosomally integrated a novel HR-NHEJ double reporter cassette. *p<0.05, ***p<0.001 of Student's t test.


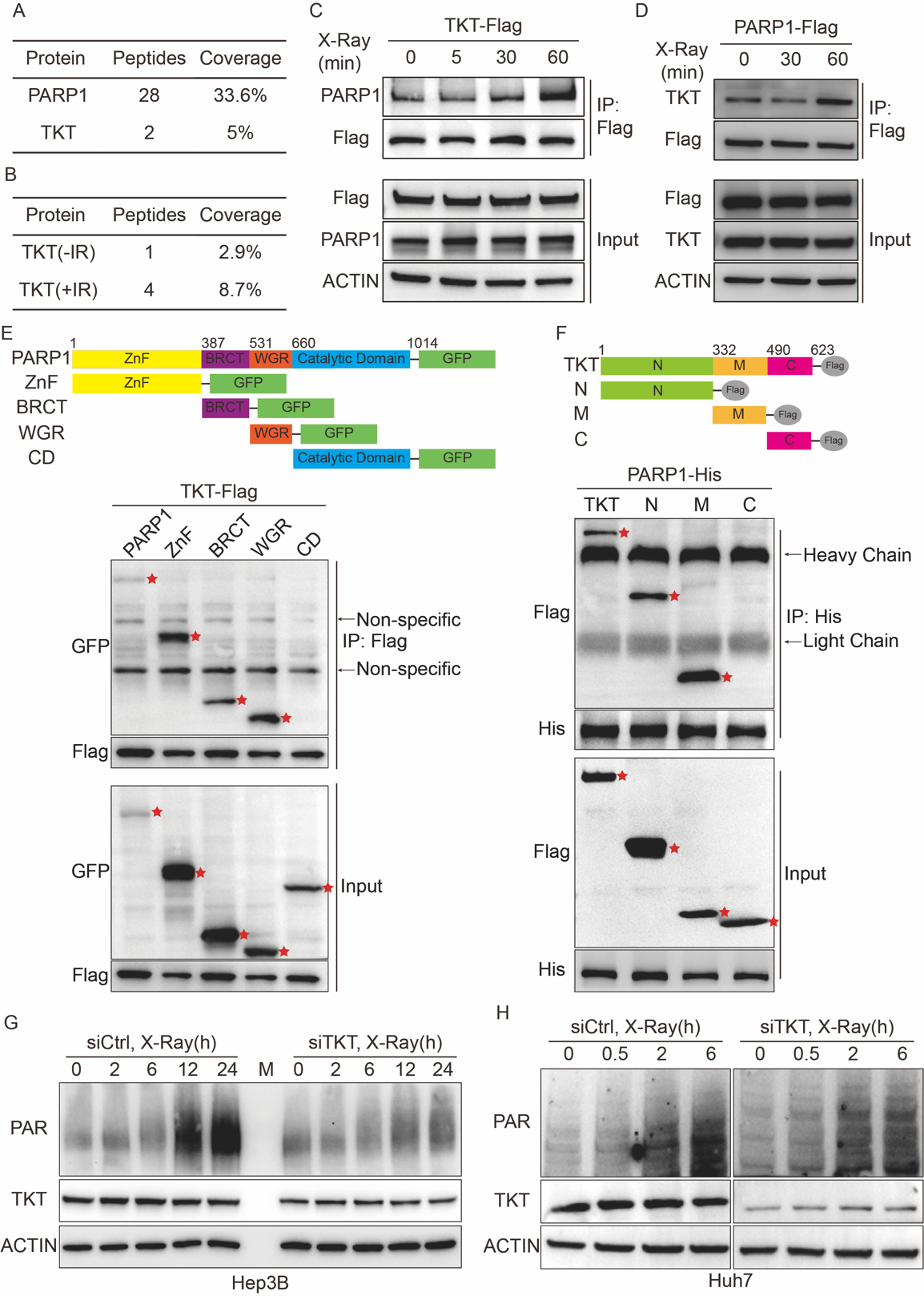


**Figure S3. The interaction between TKT and PARP1 existed in HCC and 293FT cells treated with X-Ray.** (A) The peptides and coverage of PARP1 and TKT from the IP-MS data immunoprecipitated with PARP1 antibody in HCC tissues. (B) The peptides and coverage of TKT from the IP-MS data immunoprecipitated with PAR antibody in 293FT cells in response to X-Ray. (C-D) Co-IP and WB analysis of the interaction between TKT and PARP1 in TKT-Flag transfected (C) or PARP1-Flag transfected (D) Hep3B cells at different time points after X-Ray treatment. (E-F) Schematic representation of TKT and PARP1 truncation mutants used in this study and Co-IP assays of the putative interaction domains between TKT and PARP1. (G-H) Immunoblotting detection of PARylation in siCtrl or siTKT transfected Hep3B and Huh7 cells at different time points after X-Ray treatment.


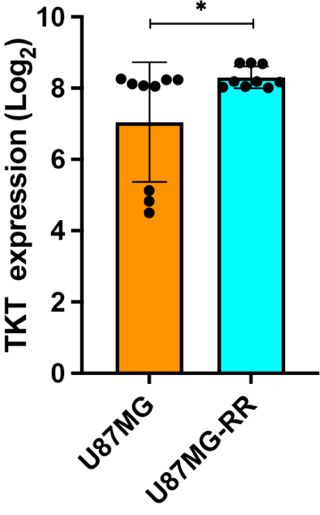


**Figure S4. The expression of TKT mRNA was upregulated in radioresistant glioblastoma cells.** The mRNA level of TKT in radioresistant glioblastoma cells. *p<0.05 of Student's t test.
